# Structural basis of signalling by TIR domain containing proteins

**DOI:** 10.1063/4.0000987

**Published:** 2025-10-27

**Authors:** Thomas Ve

**Affiliations:** Institute for Biomedicine and Glycomics, Griffith University, Goldcoast, QLD, Australia

## Abstract

The Toll/interleukin-1 receptor (TIR) domain is found in animal, plant and bacterial immune systems, and function through self-association and homotypic interactions with other TIR domains. [1]. It was first described as a protein-protein interaction module mediating signalling downstream of the Toll-like receptor (TLR) and interleukin-1 receptor (IL-1R) families in animals. However, studies of the neurodegenerative disease therapeutic target SARM1, plant immune receptors, and many bacterial TIR domain-containing proteins revealed that TIR domains have self- association-dependent enzymatic activities [2] and can produce diverse signaling molecules using nicotinamide adenine dinucleotide (NAD^+^) as a substrate. We have reconstituted TIR domain signalosomes and characterised them using an integrative structural biology approach combining MicroED, cryoEM, NMR, site directed mutagenesis, cellular assays and chemical tools. Our results have provided structural insight into TLR signal transduction [3-4] and revealed mechanisms of SARM1 activation, NAD+ cleavage and inhibition [5-6] as well as chemical structures of novel immune signalling molecules and their role in bacterial antiphage defence systems [7-8]. Collectively, our studies have led to new models for signal transduction in response to infection and provided rational avenues for the design of new therapeutics to combat a range of neurodegenerative diseases.

## References

[c1] Li et al (2023) Curr Opin Microbiology 74, 102316 10.1016/j.mib.2023.102316 37084552

[c2] Horsefield et al (2019) Science 365, 793 10.1126/science.aax1911 31439792

[c3] Ve et al (2017) Nat Struct Mol Biol 24, 743 10.1038/nsmb.3444 PMC8059215 28759049

[c4] Clabbers et al (2021) Nat Commun 12, 2578 10.1038/s41467-021-22590-6 PMC8110528 33972532

[c5] Figley et al (2021) Neuron 79, 1118 10.1016/j.neuron.2021.02.009 PMC8174188 33657413

[c6] Shi et al (2022) Mol Cell 82, 1643 10.1016/j.molcel.2022.03.007 PMC9188649 35334231

[c7] Manik et al (2022) Science, eadc8969 36048923 10.1126/science.adc8969 PMC13025242

[c8] Shi et al (2024) Sci Adv, eadn3310 10.1126/sciadv.adn3310 PMC11204291 38924412

